# A Large-Scale Building Unsupervised Extraction Method Leveraging Airborne LiDAR Point Clouds and Remote Sensing Images Based on a Dual P-Snake Model

**DOI:** 10.3390/s24237503

**Published:** 2024-11-25

**Authors:** Zeyu Tian, Yong Fang, Xiaohui Fang, Yan Ma, Han Li

**Affiliations:** 1 State Key Laboratory of Geo-Information Engineering, Xi’an Research Institute of Surveying and Mapping, Xi’an 710054, China; tianzeyu@hljit.edu.cn; 2 College of Surveying and Mapping, Heilongjiang Institute of Technology, Harbin 150050, China; fangxiaohui@hljit.edu.cn (X.F.); mayan@hljit.edu.cn (Y.M.); 3 College of Computer Science and Technology, Harbin Engineering University, Harbin 150801, China; 840163997@hrbeu.edu.cn

**Keywords:** building extraction, LiDAR point cloud, remote sensing image, dual P-snake model

## Abstract

Automatic large-scale building extraction from the LiDAR point clouds and remote sensing images is a growing focus in the fields of the sensor applications and remote sensing. However, this building extraction task remains highly challenging due to the complexity of building sizes, shapes, and surrounding environments. In addition, the discreteness, sparsity, and irregular distribution of point clouds, lighting, and shadows, as well as occlusions of the images, also seriously affect the accuracy of building extraction. To address the above issues, we propose a new unsupervised building extraction algorithm PBEA (Point and Pixel Building Extraction Algorithm) based on a new dual P-snake model (Dual Point and Pixel Snake Model). The proposed dual P-snake model is an enhanced active boundary model, which uses both point clouds and images simultaneously to obtain the inner and outer boundaries. The proposed dual P-snake model enables interaction and convergence between the inner and outer boundaries to improve the performance of building boundary detection, especially in complex scenes. Using the dual P-snake model and polygonization, this proposed PBEA can accurately extract large-scale buildings. We evaluated our PBEA and dual P-snake model on the ISPRS Vaihingen dataset and the Toronto dataset. The experimental results show that our PBEA achieves an area-based quality evaluation metric of 90.0% on the Vaihingen dataset and achieves the area-based quality evaluation metric of 92.4% on the Toronto dataset. Compared with other methods, our method demonstrates satisfactory performance.

## 1. Introduction

Automatic large-scale building extraction is crucial for environmental monitoring, disaster management [1,2], urban planning [3], cultural heritage protection, and land use planning [4,5], and it has emerged as a significant research topic in geoinformatics and remote sensing [6]. Building extraction methods are mainly divided into three categories, including extraction methods based on the images, extraction methods based on point clouds, and extraction methods based on point clouds and images.

### 1.1. The Building Extraction Methods Based on the Images

The multi-scale image segmentation algorithms [7], image edge detection algorithms, and image region growing algorithms [8,9] can effectively extract building boundaries from optical images. But the extraction accuracies and reliabilities of these methods are often hindered by the lighting, shadows, and occlusion of the images [10].

To address these issues, Reda et al. proposed an improved edge region convolutional structure to enhance the accuracy of the building detection, which is more resilient to shadows [11]. Raghavan et al. introduced an extended convolutional structure for pixel-level building detection and demonstrated its improved performance under varying lighting conditions and structural influences [12]. Protopapadakis E et al. developed a deep neural network (DNN) framework powered by stacked autoencoders (SAEs) and semi-supervised learning (SSL), which can achieve high-precision building extraction [13]. Zhang et al. utilized a combination method of the centroid clustering and contour analysis to automatically extract buildings [14]. Although these methods have made some improvements in addressing environmental factors, the texture complexity of optical images still interferes with boundary differentiation and regularization [15,16]. This can lead to lower accuracy and completeness in building extraction in many practical applications [17].

### 1.2. The Building Extraction Methods Based on the Point Clouds

Due to the high precision 3D information of the point clouds, the building extraction methods based on the point clouds can effectively mitigate the impact of the lighting, shadows, and occlusions of the images on the extraction accuracy and completeness [18,19].

But these building extraction methods based on point clouds also face challenges. For example, the discreteness, sparsity, and irregular distribution of point clouds limit the efficacy of these extraction methods in high-density, highly complex urban environments [20,21].

To address these challenges, Feng et al. proposed an improved minimum bounding rectangle (IMBR) algorithm to accurately extract regularized building boundaries from ALS point clouds, even in partially occluded scenarios [22]. Santos et al. introduced an excellent iterative CD-spline (Curvature-Degree) regularization method, which can automatically select the appropriate polynomial function for each section of a building’s roof boundary [23]. Awrangjeb et al. proposed a novel segmentation technique to extract a building, which has higher building detection accuracy and roof plane extraction in complex urban scenes [24]. Yang et al. used a marked point processing method to extract building outlines from ALS point clouds and validated its effectiveness using the standard dataset provided by ISPRS [25]. Ozdemir et al. have also developed an automatic building outline regularization (ABORE) method with superior performance, which can generate 2D orthogonal building boundaries from irregular building footprint polygons using raw point clouds [26]. Widyaningrum et al. proposed a robust geometric information extraction method based on the medial axis transform (MAT), which can detect building corner points and connect these points to form the building boundary polygons [27]. Liu K et al. devised an innovative building extraction method using the minimum cut approach, which can accurately extract most buildings, even those with curved roofs [28]. Dey E K et al. introduced an improved building detection and roof plane extraction method using a robust outlier detection algorithm and plane-fitting algorithm; it shows low sensitivity to outliers and is unlikely to generate spurious planes [29]. Hui Z et al. proposed a multi-constraints graph segmentation building extraction method, and this method can extract buildings based on objects to improve the completeness of building extraction [30].

### 1.3. Building Extraction Methods Based on Point Clouds and Images

To further improve the accuracy of building extraction, recent studies have focused on how to leverage the advantages of both point clouds and images simultaneously. Wang et al. proposed a multi-stage urban building extraction method based on dual-temporal spectral, height, and corner point information, which effectively combines the advantages of images and point clouds [31]. Siddiqui et al. proposed a new gradient-based extraction (GBE) method by converting height information into intensity images, which can effectively remove trees and extract buildings of various sizes [32]. Nguyen et al. introduced a new super-resolution snake model (SRSM) that can achieve excellent building extraction results in large-scale urban buildings [33]. Ramiya et al. used a region growing algorithm and multiple classifier system to automatically detect buildings, and validated the model on the ISPRS benchmark dataset [34]. Gilani et al. developed a graph-based algorithm that combines multispectral images and airborne point clouds to accurately delineate building boundaries [35]. Chen et al. developed an innovative automatic building extraction method that employs adaptive iterative segmentation and hierarchical overlay analysis on point clouds and high-spatial-resolution images [36]. Mousa Y A et al. introduced a combination of data-driven and model-driven methods for building boundary regularization [37]. Maltezos et al. augmented raw LiDAR data using physical features and proposed a deep learning paradigm to extract buildings [38]. Zhang et al. proposed a hybrid attention-aware fusion network (HAFNet) for building extraction, which can adaptively select and combine complementary features from imagery and LiDAR data [39].

Although these above methods have improved extraction performance to some extent, the methods have not fully utilized the advantages of point clouds and images.

In order to fully leverage the advantages of both point clouds and images, this paper proposes a new building extraction algorithm, PBEA (Point and Pixel Building Extraction Algorithm), and a new dual P-snake model (Dual Point and Pixel Snake Model). This proposed PBEA and dual P-snake model can effectively eliminate the influence of lighting, shadows, and occlusions of the images by utilizing point clouds. And this PBEA and dual P-snake model can also effectively eliminate the influence of the discreteness, sparsity, and irregular distribution of point clouds by utilizing the images. This dual P-snake model uses the building point cloud boundary as the inner boundary and uses the building image pixel boundary as the outer boundary. And this image pixel boundary is also constrained by the point cloud boundary. This dual P-snake model enables interaction and convergence between the inner and outer boundaries to achieve an accurate building boundary. Using the dual P-snake model and polygonization, this PBEA can accurately extract large-scale buildings.

The remainder of this paper is organized as follows. In Section 2, we provide a detailed description of the proposed method. Section 3 presents the experimental data and results. Finally, Section 4 offers the conclusions.

## 2. Methods

**Figure 1:**
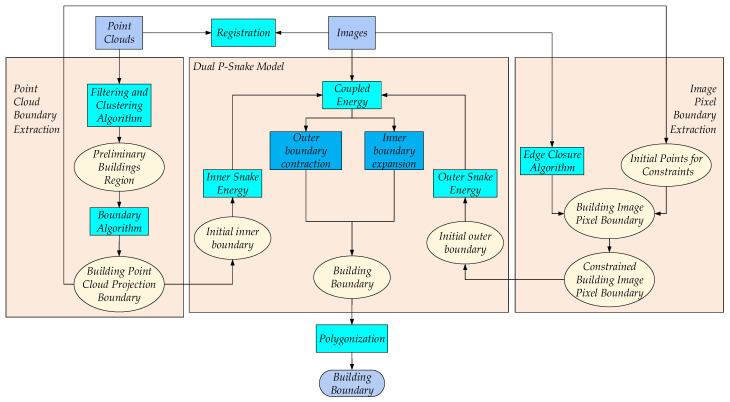
Flow chart of PBEA.

The proposed dual P-snake model (Dual Point and Pixel Snake Model) and PBEA (Point and Pixel Building Extraction Algorithm) are illustrated in Figure 1. Firstly, we performed point cloud and image registration. Secondly, we removed the ground points from the point clouds, clustered the building, and extracted the building point cloud boundary. And we projected this point cloud boundary onto the corresponding image to obtain the point cloud projection boundary $A_{1}^{img}$. Thirdly, we extracted the building image pixel boundary from the image. Then, we constrained this image pixel boundary to the point cloud projection boundary $A_{1}^{img}$ to obtain the constrained image pixel boundary $B_{2}^{img}$. Fourthly, the point cloud projection boundary $A_{1}^{img}$ and constrained image pixel boundary $B_{2}^{img}$ served as the inner and outer boundaries of the dual P-snake model. This dual P-snake model can effectively control the co-evolution of the inner and outer boundaries to accurately form the building boundary using a new coupled energy $E_{coupled}$. Finally, using the dual P-snake model and polygonization, the PBEA can effectively extract large-scale buildings. The above steps are provided in detail the subsequent text.

### 2.1. Registration

Initially, the camera’s intrinsic parameters (including focal length and optical center coordinates) and extrinsic parameters (including the camera’s rotation and translation matrices) are needed. These parameters enable transformation of the points from the 3D world coordinate system to the camera coordinate system. Using the extrinsic matrix, we transformed the point cloud coordinates to align them with the camera coordinate system. This transformation is achieved by applying rotation and translation transformations to each point. By using the camera intrinsic parameter matrix, we projected the 3D point cloud located in the camera coordinate system onto the 2D image plane, and converted the 3D coordinates into pixel coordinates. This projection process follows the perspective projection formula [40]. We implemented the above registration process by using the commercial software Visual Studio 2019 (Microsoft Corp., Redmond, WA, USA) along with the open-source computer vision library OpenCV 4.9.0 (Intel Corp., Santa Clara, CA, USA).

### 2.2. Building Point Cloud Boundary Extraction

**Figure 2:**
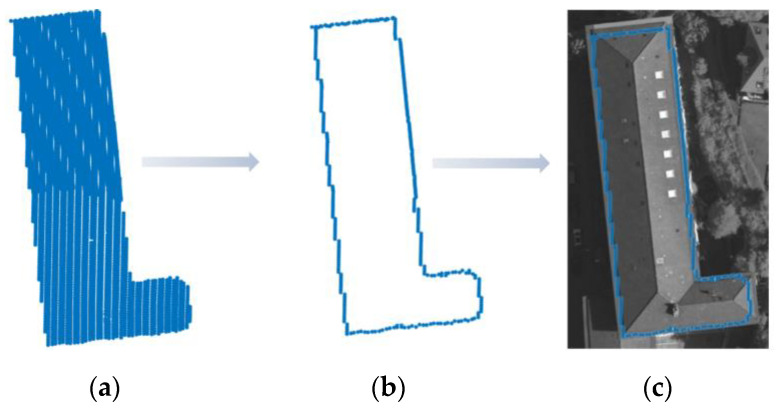
Building boundary extraction from the point clouds: (**a**) Cluster results of FEC algorithm. (**b**) Boundary extracted by the boundary algorithm. (**c**) Projection onto the image.

The building point cloud boundary extraction is shown in Figure 2. Firstly, we removed the ground points from the point clouds by robust filtering [41], which was implemented in the commercial software package SCOP++ 5.6 (Trimble Navigation Co. Ltd., Sunnyvale, CA, USA). Next, we applied the FEC algorithm [42] to cluster the buildings. Then, we used the boundary algorithm [43] to extract the boundary points from the point clouds, and these boundary points are represented as the building point cloud boundary point set $A_{1}^{poi}$. Finally, the extracted building point cloud boundary point set $A_{1}^{poi}$ were projected onto the corresponding image to yield the building point cloud projection boundary point set $A_{1}^{img}$. This point set $A_{1}^{img}$ can serve as the initial inner boundary of the dual P-snake model and can also be used to constrain and denoise the results of the edge closure algorithm [44]. 

### 2.3. Building Image Pixel Boundary Extraction

We used the edge closure algorithm [44] to extract the building image pixel boundary $B_{1}^{img}$ from the image. The edge closure algorithm can address the issue of discontinuities in Canny [8] edges at intersections and can reduce the occurrence of boundary gaps. But the edge closure algorithm is highly susceptible to noise.

**Figure 3:**
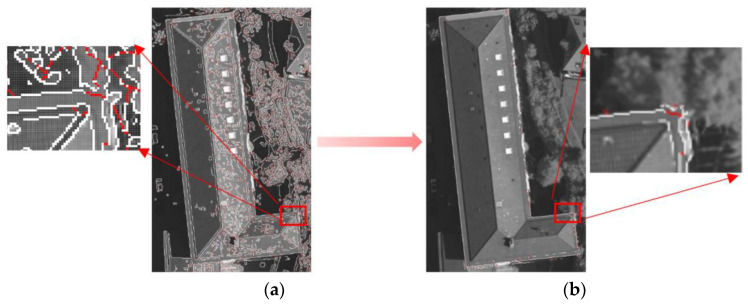
Building boundary extracted from the image: (**a**) Building boundary extracted by the edge closure algorithm, where white lines represent the canny edges and red lines indicate the extended closed edges. (**b**) Building boundary constrained by the point cloud projection boundary.

To address this, we used the building point cloud projection boundary point set $A_{1}^{img}$ to construct the constraint range. We only retained the pixel points extracted by the edge closure algorithm within the constraint range. All other pixel points extracted by the edge closure algorithm were removed. Through experiments, the constraint range was set to a threshold of 5 pixels outward from each point of the point set $A_{1}^{img}$. This method can effectively constrain and denoise the image pixel boundary points extracted by the edge closure algorithm, and these boundary points are represented as the constrained building image pixel boundary point set $B_{2}^{img}$. The building boundary extracted by the edge closure algorithm and the building boundary constrained by the point cloud projection boundary are as shown in Figure 3.

In the subsequent processing, the constrained building image pixel boundary point set $B_{2}^{img}$ can serve as the initial outer boundary of the dual P-snake model and work in conjunction with the initial inner boundary to drive the dual P-snake model.

### 2.4. Building Boundary Extraction

#### 2.4.1. Traditional Snake Model

The traditional snake model, also known as the active boundary model, is a dynamic curve x(s)=(x(s),y(s)), where s∈[0,1] is the normalized arc length. It is defined within an image domain and can deform under the influence of internal and external forces [45]. Mathematically, the behavior of the snake is governed by an energy function defined as follows:

(1) $$E_{snake}=\int_{0}^{1}\left(E_{int}\left(x\left(s\right)\right)+E_{ext}\left(x\left(s\right)\right)\right)ds$$

(2) $$E_{int}\left(x\left(s\right)\right)=\frac{1}{2}\left(\alpha {\left|\frac{\partial x}{\partial s}\right|}^{2}+\beta {\left|\frac{\partial^{2}x}{\partial s^{2}}\right|}^{2}\right)$$

(3) $$E_{ext}\left(x\left(s\right)\right)=E_{img}\left(x\left(s\right)\right)+E_{con}\left(x\left(s\right)\right)$$

Here, $E_{int}$ represents the internal energy term, and $E_{ext}$ represents the external energy term. The internal energy term is related to the stretching and curvature of the snake and is controlled by the weighting parameters α and β. Smaller values of α and β result in a shorter and smoother boundary. The external energy $E_{ext}$ is composed of forces generated by the image itself, $E_{img}$, and other constraint forces, $E_{con}$. The external image energy $E_{img}$ is associated with the prominent features of the image, such as lines, edges, and terminations (i.e., line segment endpoints, corners). Mathematically, it is expressed as follows:

(4) $$E_{img}=w_{line}E_{line}+w_{edge}E_{edge}+w_{term}E_{term}$$

Here, $w_{line}$, $w_{edge}$, and $w_{term}$ are the weights for the respective prominent features [45].

By minimizing the energy function, we can obtain the parametric curve. This parametric curve is called the snake boundary. This numerical solution of minimizing the energy function can be obtained through discretization and iterative solution. This numerical solution is described in detail in [45].

#### 2.4.2. GVF Snake Model

Based on the traditional snake model, the Gradient Vector Flow (GVF) and GVF snake [46] allow for more flexible initialization, encourage convergence to boundary concavities, and enhance robustness. The GVF is added to the snake energy function as the external constraint force, which can guide the snake boundary toward or away from a particular feature. The GVF field is defined as the vector field v(x, y)=(u(x, y),v(x, y)) that minimizes the energy function, as follows:

(5) $$E_{GVF}=\iint \mu \left(u_{x}^{2}+u_{y}^{2}+v_{x}^{2}+v_{y}^{2}\right)+{\left|\nabla f\right|}^{2}{\left|v-\nabla f\right|}^{2}dxdy$$

Here, μ is a controllable smoothness term, and f represents the external force [46].

The solution method of minimizing the energy function is similar to the traditional snake model and is described in detail in [46]. The parametric curve minimizing the energy function is called the GVF snake boundary.

#### 2.4.3. Dual P-Snake Model

Based on the GVF snake model, we propose a new snake model: dual P-snake model (Dual Point and Pixel Snake Model). In the dual P-snake model, we employ the GVF snake on the initial inner and outer boundaries. The initial inner boundary is the building point cloud projection boundary point set $A_{1}^{img}$. The initial outer boundary is the constrained building image pixel boundary point set $B_{2}^{img}$. The point sets $A_{1}^{img}$ and $B_{2}^{img}$ serve as the input of this dual P-snake model. And in the dual P-snake model, we introduce a new coupled energy $E_{coupled}$. This coupled energy $E_{coupled}$ can promote the co-evolution of the inner and outer boundaries to form a dual P-snake boundary. This dual P-snake boundary can accurately fit the real building outline.

Additionally, we incorporate an additional inflation term [47] (balloon model) into the initial inner boundary of the dual P-snake model as an external constraint force. This term can cause the initial inner boundary to expand outward. This term is as follows:

(6) $$F_{balloon}=k\times \vec{n}\left(s\right)$$

Here, k represents the force magnitude, and $\vec{n}\left(s\right)$ is the normal vector to the curve [47]. This balloon model can simulate the balloon inflation by continuously pushing the inner boundary points outward. This balloon model can control the inner boundary to expand rather than shrink.

We introduce a new coupled energy $E_{coupled}$ to control the interaction between the inner and outer boundaries as an external constraint force. The coupled energy $E_{coupled}$ achieves the purposes of outward expansion of the initial inner boundary and inward contraction of the initial outer boundary. By integrating the interaction between the inner and outer boundaries, the dual P-snake model can significantly enhance the accuracy and robustness of the building extraction.

The proposed coupled energy $E_{coupled}$ consists of two constraint components. The first component is the centroid constraint component $E_{centroid}$ and second component is the Hausdorff constraint component $E_{Hausdorff}$. The coupled energy $E_{coupled}$ is expressed as follows:

(7) $$E_{coupled}={{\gamma \times E}_{centroid}+\tau \times E}_{Hausdorff}$$

γ and τ are the weighting parameters to control the centroid constraint component $E_{centroid}$ and Hausdorff constraint component $E_{Hausdorff}$.

The centroid constraint component $E_{centroid}$ can control the alignment of the inner boundary and outer boundary as a whole. The centroid constraint component $E_{centroid}$ is expressed as follows:

(8) $$E_{centroid}=1-exp\left(-\frac{{\left\|c_{inner}-c_{outer}\right\|}^{2}}{\delta_{c}}\right)$$

$c_{inner}$ is the centroid of the inner boundary, $c_{outer}$ is the centroid of the outer boundary and $\delta_{c}$ is a scale factor.

The Hausdorff constraint component $E_{Hausdorff}$ can maintain the same shapes of the inner boundary and outer boundary, and it can control the alignment of the inner boundary and outer boundary in detail. The mathematical formulation is as follows:

(9) $$E_{Hausdorff}=1-exp\left(-\frac{d_{H}^{bi}{\left(A_{1}^{img},B_{2}^{img}\right)}^{2}}{\delta_{H}}\right)$$

where $d_{H}^{bi}$ is the bidirectional Hausdorff distance [48], $\delta_{H}$ is a scale factor, $A_{1}^{img}$ is the inner boundary, and $B_{2}^{img}$ is the outer boundary.

The energy function of the dual P-snake model is expressed as follows:

(10) $$E_{P\text{-}snake}=E_{inner}+E_{outer}$$

$E_{inner}$ is the inner energy function of the GVF snake on the inner boundary, and $E_{outer}$ is the outer energy function of the GVF snake on the outer boundary. The balloon model $F_{balloon}$ is introduced into the energy function $E_{inner}$ as an external constraint force. And the proposed coupled energy $E_{coupled}$ is introduced into the energy function $E_{outer}$ as an external constraint force.

The energy function $E_{P\text{-}snake}$ can be minimized by the discretization and iterative solution, which is described in detail in [45]. By minimizing the energy function $E_{P\text{-}snake}$, we can obtain a parametric curve that can accurately match the building boundary. This parametric curve is called the P-snake boundary.

By using the dual P-snake model, we achieved interaction and convergence of the initial inner and outer boundaries into an accurate P-snake building boundary, and this P-snake boundary is represented as the building boundary point set $C_{1}^{img}$.

**Figure 4:**
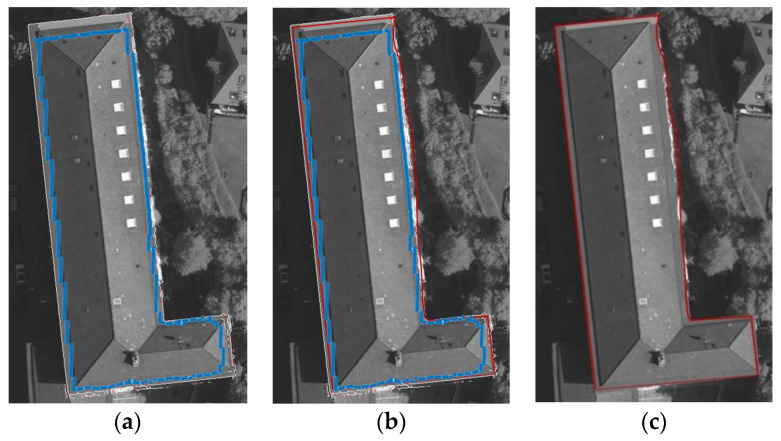
Building boundary extracted by the dual P-snake model: (**a**) Comparison of the initial inner and outer boundaries. (**b**) Comparison among the initial inner boundary, initial outer boundary, and boundary extracted by the dual P-snake model. (**c**) Boundary extracted by the dual P-snake model.

In Figure 4, the blue line represents the building point cloud projection boundary point set $A_{1}^{img}$, which is the initial inner boundary. The white line represents the constrained building image pixel boundary point set $B_{2}^{img}$, which is the initial outer boundary. And the red line represents the boundary extracted by the dual P-snake model. Figure 4a shows the inner and outer boundaries. Figure 4b compares three boundaries and indicates that the building boundary extracted by the dual P-snake model is accurate. Figure 4c independently shows the boundary extracted by the dual P-snake model for better comparison with the building’s base map.

**Figure 5:**
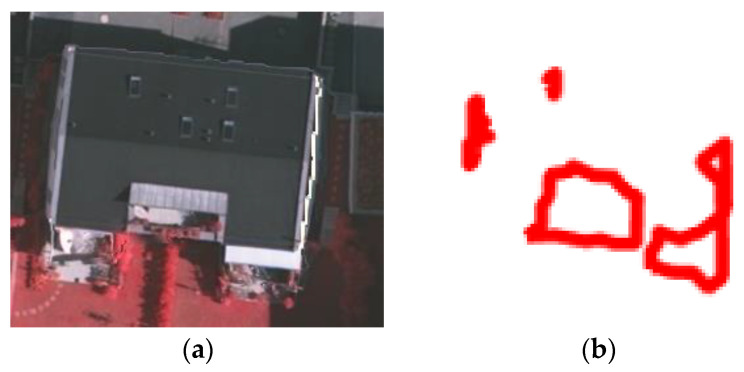

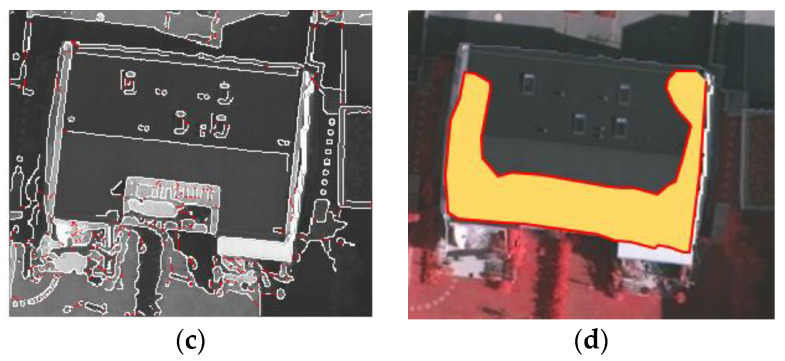
Influence of the sparse region of the point clouds on the building extraction: (**a**) Remote sensing image of the building. (**b**) Building boundary extracted from the sparse region of the point clouds. (**c**) Building boundary extracted from the image. (**d**) Building boundary extracted by the dual P-snake model.

The dual P-snake model can effectively eliminate the influence of the discreteness, sparsity, and irregular distribution of the point clouds by utilizing the images, as is shown in Figure 5. Figure 5b shows that the building boundary extracted from the sparse region of the point clouds is extremely incomplete.

By utilizing the boundary extracted from the image in Figure 5c and the incomplete boundary extracted from the point clouds in Figure 5b, the dual P-snake model can obtain the building boundary in Figure 5d. Although the dual P-snake model cannot also capture the complete building boundary in the sparse region of the point clouds, this model also greatly improves the completeness of the extracted building. The dual P-snake model can effectively reduce the influence of the sparse region of the point clouds on the completeness of the extracted building.

**Figure 6:**
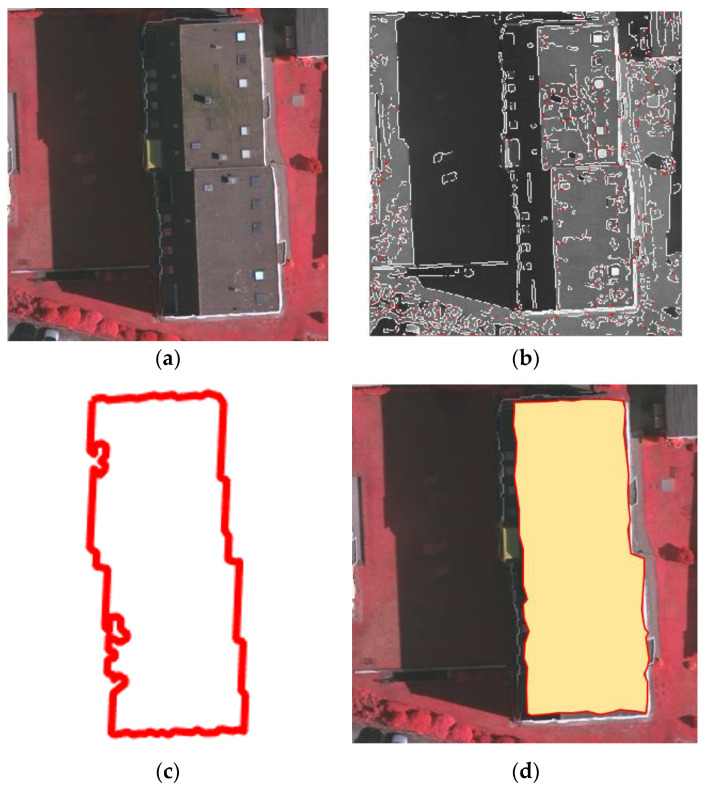
Influence of the shadow region of the image on the building extraction: (**a**) Remote sensing image of the building. (**b**) Building boundary extracted from the shadow region of the image. (**c**) Building boundary extracted from the point clouds. (**d**) Building boundary extracted by the dual P-snake model.

The dual P-snake model can effectively eliminate the influence of the lighting and shadows of the images by utilizing the point clouds, as is shown in Figure 6. Figure 6b shows that the building boundary extracted from the shadow region of the image is incomplete and may be confused with the outline of the shadow region.

By utilizing the boundary extracted from the point clouds in Figure 6c and the inaccurate boundary extracted from the image in Figure 6b, the dual P-snake model can obtain the building boundary in Figure 6d. The point cloud boundary can effectively constrain the image boundary. The dual P-snake model can capture the complete and accurate building boundary in the shadow region of the image by incorporating the point cloud boundary.

**Figure 7:**
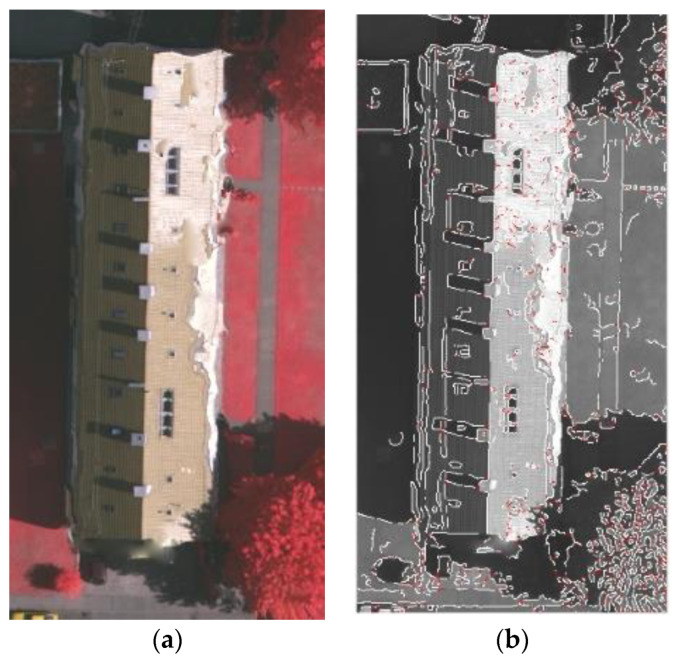

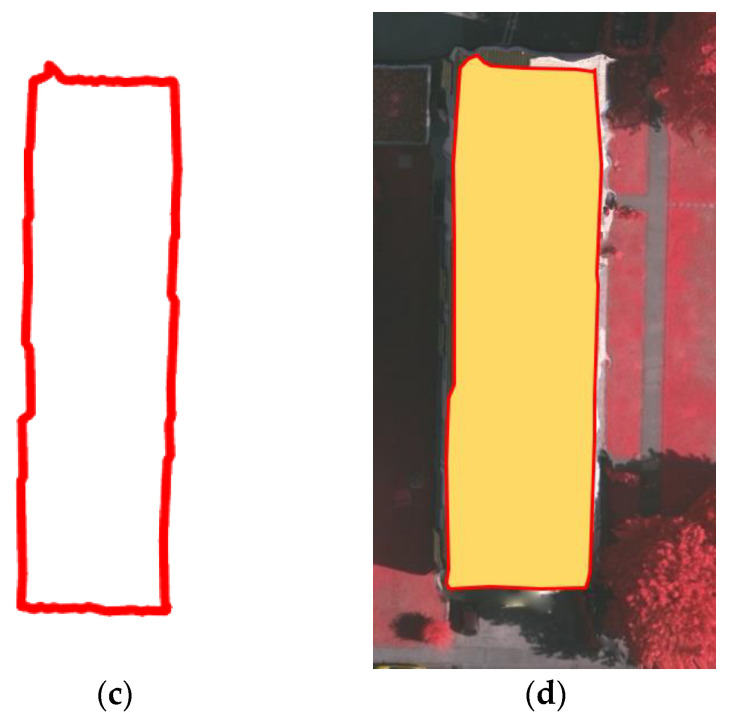
Influence of the occlusion regions of the image on the building extraction: (**a**) Remote sensing image of the building, and the lower right corner of the building is obscured by trees and shadows. (**b**) Building boundary extracted from the occlusion region of the image. (**c**) Building boundary extracted from the point clouds. (**d**) Building boundary extracted by the dual P-snake model.

The dual P-snake model can effectively eliminate the influence of the occlusions of the images by utilizing the point clouds, as is shown in Figure 7. Figure 7b shows that the lower right corner of the building boundary extracted from the occlusion region of the image is missing.

By utilizing the boundary extracted from the point clouds in Figure 7c and the missing boundary extracted from the image in Figure 7b, the dual P-snake model can obtain the building boundary in Figure 7d. The dual P-snake model can capture the complete building boundary in the occlusion region of the image by incorporating the point cloud boundary.

The dual P-snake model fully exploit the advantages of the point clouds and images to refine the building boundary.

### 2.5. Polygonization of the Building Boundary

**Figure 8:**
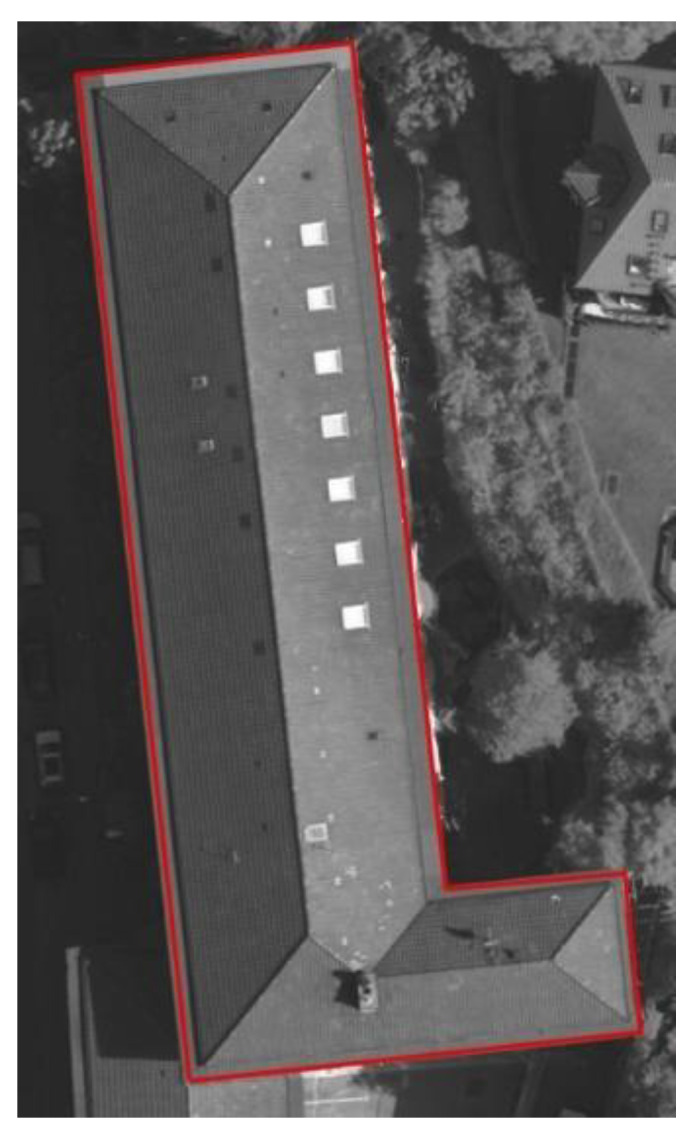
Results of the building boundary polygonization.

The building boundary point set $C_{1}^{img}$ extracted by the dual P-snake model is used for the polygonization of the building boundary. The point set $C_{1}^{img}$ was initially simplified by the Douglas–Peucker algorithm [49]. The simplified results were then polygonized by the Dutter method [50]. The final results of the building boundary polygonization are shown in Figure 8.

## 3. Results

### 3.1. Datasets

To evaluate our dual P-snake model and PBEA, we used Vaihingen and Toronto datasets provided by the International Society for Photogrammetry and Remote Sensing (ISPRS) [51]. The datasets include LiDAR point clouds and remote sensing images. The point clouds and images of the datasets have been precisely co-registered and do not require further registration processing.

#### 3.1.1. Dataset 1: Vaihingen

The Vaihingen dataset was captured in Vaihingen, Germany. This dataset includes three test areas with the true building boundary. Area 1 is the city center of Vaihingen, which is characterized by dense development and complex historical buildings along with some trees. Area 2 consists of several tall residential structures and surrounding trees. Area 3 includes small detached houses and surrounding trees.

The airborne LiDAR point clouds were acquired using the Leica ALS50 system (Leica Geosystems AG., Heerbrugg, Switzerland) on 21 August 2008. And this point cloud consists of 10 ALS strips with a field of view of 45°, an average flight altitude of 500 m, and an average point density of 4 pts/m^2^.

The digital aerial images were captured by the Intergraph/ZI DMC camera (Intergraph, Huntsville Corp., FL, USA) on 24 July and 6 August 2008, with a ground resolution of 8 cm. The camera parameters were a focal length of 120 mm, flight altitude of 900 m, and ground sampling distance of 8 cm.

In addition, ISPRS also provided the Digital Surface Model (DSM) and true orthophoto generated from the digital aerial images. And both datasets are defined on the same grid with a ground resolution of 9 cm.

#### 3.1.2. Dataset 2: Downtown Toronto

The Downtown Toronto dataset was collected from downtown Toronto, Canada, using the Microsoft Vexcel UltraCam-D (UCD) camera (Vexcel Corp., Redmond, WA, USA) and Optech airborne laser scanner ALTM-ORION M (Optech Corp., Toronto, ON, Canada). This dataset includes two test areas. Area 4 includes a mix of low-rise and high-rise buildings with different roof structures. Area 5 is a typical representation of a high-rise building cluster in a modern North American megacity.

ALS point clouds were collected by Optech’s ALTM-ORION M aircraft at a flight altitude of 650 m in February 2009. The dataset comprises six ALS strips with an average point density of 6 pts/m^2^. ISPRS also provides the DSM, which is interpolated from the ALS point clouds with a grid width of 25 cm.

The digital aerial images were captured by the UltraCam-D. These images are 7500 × 11,500 pixels in size, with a pixel size of 9 μm. The camera parameters include a focal length of 101.4 mm, flight altitude of 1600 m, and ground sampling distance of 15 cm.

The ALS point clouds and images of this dataset utilize the same coordinate system, and the relevant camera parameters are detailed in [51].

### 3.2. Performance Evaluation Metrics

Given that ISPRS provides the true building boundaries of the test areas, we utilized the following area-based evaluation metrics to assess the precision and efficacy of the building extraction methods, including quality (Q), also known as Intersection over Union (IoU) [52], completeness ($C_{p}$), and correctness ($C_{r}$) [53].

We defined the predicted building using our method as A and the true building as B. Let S(A) and S(B) denote areas of the predicted and true buildings, respectively. S(A∪B) represents the area of the union of the predicted building and true building, and $S\left(A\cap B\right)$ represents the area of the overlap between the predicted building and true building. Thus, quality can be expressed as follows:

(11) $$Q=\frac{S\left(A\cap B\right)}{S\left(A\;\cup B\right)}\times 100\%$$

where $S\left(A\cup B\right)$ is as follows:

(12) $$S\left(A\cup B\right)=S(A)+S(B)-S\left(A\cap B\right)$$

Completeness $C_{p}$ and correctness $C_{r}$ can be expressed as follows:

(13) $$C_{p}=\frac{S\left(A\cap B\right)}{S(B)}\times 100\%$$

(14) $$C_{r}=\frac{S\left(A\cap B\right)}{S(A)}\times 100\%$$

The relation formulas of Q, $C_{p}$, and $C_{r}$ are as follows:

(15) $$Q=\frac{1}{\frac{1}{C_{p}}+\frac{1}{C_{r}}-1}$$

In this study, we used the number of pixels instead of the area for the evaluation. S(A∩B) represents the number of correctly identified pixels; S(B) is the number of true pixels and includes both correctly identified and unidentified true pixels; S(A) is the number of predicted pixels and includes both correctly identified and erroneously identified pixels.

The area-based Q evaluation metric can reflect the overall accuracy of the building extraction method. The area-based $C_{p}$ evaluation metric represents the proportion of correctly identified building pixels to the total number of true building pixels. The area-based $C_{r}$ evaluation metric denotes the ratio of correct building pixels within the identified pixels. The area-based Q, $C_{p}$, and $C_{r}$ evaluation metrics achieve their best values at 100% and their worst at 0%.

Unlike the above area-based Q, completeness, and correctness evaluation metrics, we also focused on whether the building objects were correctly identified and extracted to calculate the object-based quality, completeness, and correctness evaluation metrics. If the extracted building covered at least 50% of the area of the true building, this building was considered to have been identified. Otherwise, this building was considered to have not been identified. We assessed all building objects within the region and specifically those with an area greater than 50 m^2^.

Additionally, we used the Root Mean Square Error (RMSE) as the geometric evaluation metric. This evaluation metric indicates the accuracy of the extracted building boundary relative to the true boundary. A smaller RMSE signifies better geometric precision. This evaluation method is detailed in [54].

### 3.3. Snake Model Comparison

We compared the building boundary extraction performance of different snake models across three areas in the ISPRS Vaihingen dataset. These snake models for comparison include the basic snake [45], GVF snake [46], T-snake [55], Nguyen snake [33], and our dual P-snake model. Except for our dual P-snake model, all other snake models were initialized using the building point cloud projection boundary point set.

**Figure 9:**
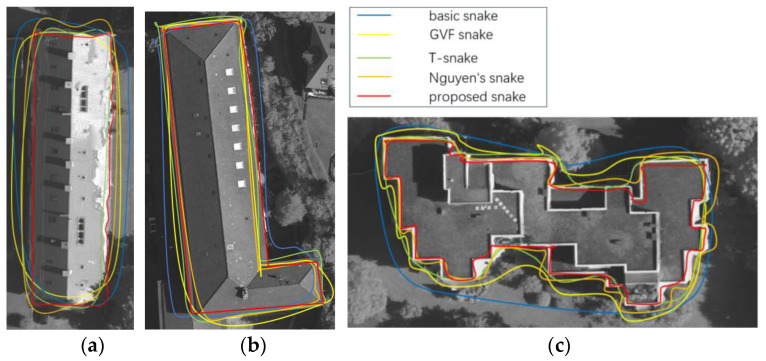
Performance of the snake models on buildings with varying complexity: (**a**) Simple rectangular building. (**b**) L-shaped building. (**c**) Building with the complex boundary.

As shown in Figure 9, our dual P-snake model consistently better converges to the true building boundary across all presented buildings. Notably, our model excels in handling the complex and prominent boundary and can converge more effectively towards building corners and concave edges. Moreover, our snake model outperforms other methods in addressing the rounded corner issue of building boundary extraction.

Table 1 summarizes the area-based average Q evaluation metrics of the building boundary extraction results of different snake models. The area-based average Q evaluation metric of our dual P-snake model is 89.1%, which is higher than the average Q evaluation metrics of all other models. The comparisons of the area-based average Q evaluation metrics demonstrate that our model outperforms all other snake models.

### 3.4. Building Extraction Experiment

#### 3.4.1. Performance on the Vaihingen Dataset

**Figure 10:**
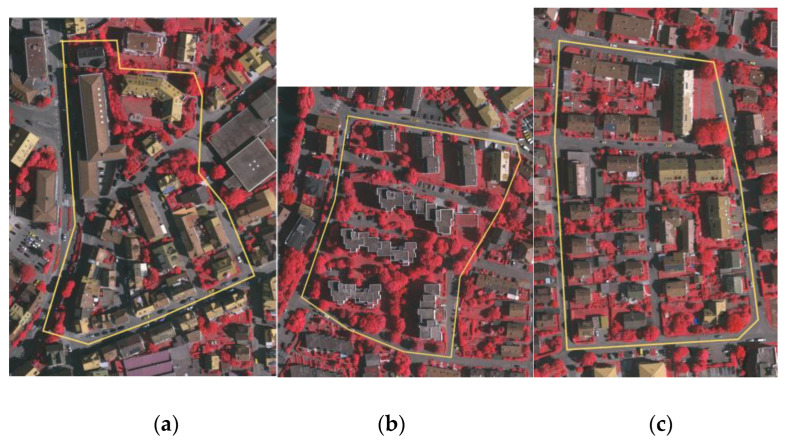

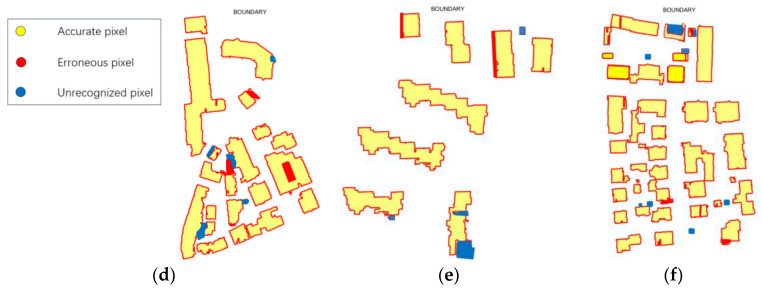
Buildings extracted from the Vaihingen dataset by our PBEA: (**a**) Area 1, (**b**) Area 2, (**c**) Area 3, (**d**) extraction results of Area 1, (**e**) extraction results of Area 2 (**f**) extraction results of Area 3.

We applied the proposed PBEA to three test areas in the ISPRS Vaihingen dataset. The visualization results without the polygonization operation are shown in Figure 10. Yellow regions represent the correctly identified building pixels, red regions represent the incorrectly identified building pixels, and blue regions represent the unidentified building pixels. Although the majority of the building boundaries were well extracted, the performance of the boundary extraction was poor on the sparse regions of the point clouds, and there were also misidentified pixels between closely adjacent buildings.

Currently, the most authoritative and widely used evaluation metric is the area-based quality evaluation metric. Therefore, we compare our PBEA with the top five methods in terms of the area-based average quality evaluation metric ranking list on the ISPRS Vaihingen dataset. This ranking list is from the website of the ISPRS Test Project on Urban Classification and 3D Building Reconstruction [56]. The area-based average quality evaluation metric is the average of the area-based quality evaluation metrics of three test areas on the Vaihingen dataset. Additionally, we also compared our PBEA with 10 state-of-the-art methods from published studies in terms of the area-based average quality evaluation metric. We used the area-based quality evaluation metric as the primary evaluation metric, and also supplemented the object-based evaluation and Root Mean Square Error (RMSE) metrics in the comparison.

In Table 2, we compare our PBEA with the top five methods on the ISPRS Vaihingen dataset in terms of the area-based evaluation metrics, and C_p_-Are, C_r_-Are, and Q-Are represent the area-based completeness, correctness, and quality evaluation metrics. In addition, bold font represents the best performance, underline represents the second-best performance, and wavy line represents the third-best performance.

The comparison results in Table 2 show that our PBEA achieves higher precision than the other top five methods on the most important average quality evaluation metric, and our PBEA also achieves the third-best performance on the average completeness and correctness evaluation metrics. Our method does not achieve optimal performance on the average completeness and correctness evaluation metrics, mainly due to the sparse regions in the point clouds causing severe boundary loss. Although our method can supplement these sparse regions in the point clouds using images, the supplementary effect is limited. Nevertheless, our method achieves the best performance on the average quality evaluation metric, which indicates that our method is the best in overall accuracy and effectiveness.

In Table 3, we compare our PBEA with the top five methods on the ISPRS Vaihingen dataset in terms of the object-based evaluation metric; C_p_-Obj, C_r_-Obj, and Q-Obj represent the object-based completeness, correctness, and quality evaluation metrics across all building objects, and C_p50m_^2^-Obj, C_r50m_^2^-Obj, and Q_50m_^2^-Obj represent the object-based completeness, correctness, and quality evaluation metrics across these building objects with an area exceeding 50 m^2^. The differences between two types of evaluation metrics highlight the challenge that is posed by undetected small buildings.

The comparison results in Table 3 show that our PBEA achieves the best performance on one metric, second-best performance on four metrics, and third-best performance on one metric among the six object-based evaluation metrics. Compared with the other top five methods, our method is not optimal and is at a mid-range level on the object-based evaluation metrics. This is because these object-based evaluation metrics focus more on the completeness of the building. The inner boundary of our method extracted from the sparse region of the point clouds is incomplete. The incomplete inner boundary can deem our method not optimal on the object-based evaluation metrics. But our method is optimal on the area-based quality evaluation metric, and the overall advantages of our method remain evident.

Table 4 clearly shows the ranking positions of the average evaluation metrics of our method across all 42 methods. These 42 methods are from the ranking list from the website of the ISPRS Test Project on Urban Classification and 3D Building Reconstruction [56]. In Table 4, Q-Are, Q-Obj, Q_50m_^2^-Obj, and RMSE represent the average area-based quality, average object-based quality, average object-based quality with an area exceeding 50 m^2^, and average RMSE evaluation metrics.

The average area-based quality evaluation metric of our method is 90%, ranking first. The average RMSE evaluation metric of our method is 0.8, ranking third. The average object-based quality evaluation metric of our method is 83.0%, ranking third. The average object-based quality evaluation metric of our method with an area exceeding 50 m^2^ is 99.4%, ranking second.

In Table 5, we compare our PBEA with 10 state-of-the-art methods from published studies in terms of the area-based average quality evaluation metric. The 10 methods include 4 deep learning methods and 6 unsupervised methods. The comparison results in Table 5 show that our PBEA achieves the best performance on the area-based average quality evaluation.

Based on the comparison results in Table 2 and Table 5, the highest average area-based quality evaluation metric of 89.8% among all 15 comparison methods is inferior to the average area-based quality evaluation metric of 90.0% achieved by our PEBA method. In summary, our method can achieve satisfactory building extraction performance.

#### 3.4.2. Performance on the Downtown Toronto Dataset

**Figure 11:**
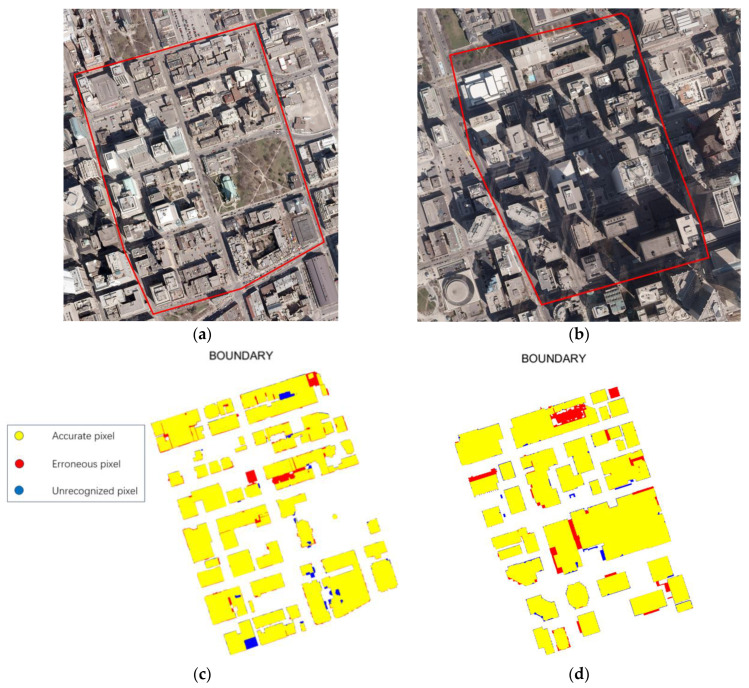
Buildings extracted from the Toronto dataset by our PBEA: (**a**) Area 4, (**b**) Area 5, (**c**) extraction results of Area 4, (**d**) extraction results of Area 5.

We applied our PBEA to two test areas of the Downtown Toronto dataset. The visualization results without the polygonization operation are shown in Figure 11. Yellow regions represent the correctly identified building pixels, red regions represent the incorrectly identified building pixels, and blue regions represent the unidentified building pixels. Our PBEA demonstrates excellent performance across the two test areas.

We compared our PBEA with the top five methods in terms of the area-based average quality evaluation metric ranking list on the ISPRS Toronto dataset. This ranking list is from the website of the ISPRS Test Project on Urban Classification and 3D Building Reconstruction [56].

In Table 6, we compare our PBEA with the top five methods on the ISPRS Toronto dataset in terms of the area-based evaluation metric. The meanings of the symbols in Table 6 are the same as those in Table 2.

The comparison results in Table 6 show that our PBEA achieves the best performance on the average quality and correctness evaluation metrics, and our PBEA also achieves the second-best performance on the average completeness evaluation metric.

In Table 7, we compare our PBEA with the top five methods on the ISPRS Toronto dataset in terms of the object-based evaluation metrics. The meanings of the symbols in Table 7 are the same as those in Table 3.

The comparison results in Table 7 show that our PBEA achieves the best performance on three metrics, second-best performance on two metrics, and third-best performance on one metric among the six object-based evaluation metrics. Compared with the other top five methods, our method is optimal on the object-based evaluation metrics.

Table 8 clearly shows the ranking positions of the average evaluation metrics of our method across all 11 methods. These 11 methods are from the ranking list on the website of the ISPRS Test Project on Urban Classification and 3D Building Reconstruction [56]. The meanings of the symbols in Table 8 are the same as those in Table 4.

The average area-based quality evaluation metric for our method is 92.4%, ranking first. The average RMSE evaluation metric of our method is 0.8, ranking first. The average object-based quality evaluation metric of our method is 88.5%, ranking first. The average object-based quality evaluation metric of our method with an area exceeding 50 m^2^ is 92.1%, ranking second.

Based on the comparison results in Table 6, Table 7 and Table 8, our method comprehensively outperforms other top five methods on the ISPRS Toronto dataset.

Through experiments on ISPRS Vaihingen and Toronto datasets, it is demonstrated that our PBEA has the ability to extract buildings with high accuracy in different regions.

Furthermore, by comparing Table 2, Table 3 and Table 4 with Table 6, Table 7 and Table 8, our PEBA performs significantly better on the Toronto dataset compared to the Vaihingen dataset. This is because the average point density of 6 pts/m^2^ in the Toronto dataset is denser than the average point density of 4 pts/m^2^ in the Vaihingen dataset. Our PBEA relies on the density of the point clouds. The sparse region of the point clouds can seriously hinder building boundary extraction. Even if our method utilizes the image boundary as a supplement, the sparse region of the point clouds can still hinder the acquisition of optimal results. Additionally, when multiple buildings are adjacent, our method tends to identify them as a single structure. These issues lead to our method not achieving optimal results on all evaluation metrics. However, our method excels in the most widely used area-based quality evaluation metric.

## 4. Discussion and Conclusions

During the process of building extraction, the building boundary extracted from the point clouds often shrinks inward relative to the actual building outline. In addition, the discrete, sparse, and irregularly distributed point clouds can also result in incomplete boundaries. The building boundary extracted from the images is often inaccurate and incomplete due to the influence of the lighting, shadows, and occlusions.

To address the above issues, we propose a new dual P-snake model (Dual Point and Pixel Snake Model) in this paper, which uses both point clouds and images simultaneously. The dual P-snake model uses the building point cloud projection boundary as the initial inner boundary and uses the constrained building image pixel boundary as the initial outer boundary. And we introduce a new coupled energy $E_{coupled}$, which can effectively control the inner boundary to expand outward, and which can control the outer boundary to converge inward. This coupled energy can effectively control the co-evolution of the inner and outer boundaries to form a P-snake boundary that can accurately fit the real building outline.

Differently from previous snake models, the proposed dual P-snake model can utilize the advantages of both point clouds and images. This dual P-snake model can effectively eliminate the influence of lighting, shadows, and occlusions of the images by utilizing the point clouds, and it can effectively eliminate the influence of the discreteness, sparsity, and irregular distribution of the point clouds by utilizing the images. And our dual P-snake model is compared with four other excellent snake models on the ISPRS Vaihingen dataset. The experimental results show that our model has the best building extraction performance and outperforms the other snake models.

Using the dual P-snake model and polygonization, we propose a new large-scale building extraction algorithm, the PBEA (Point and Pixel Building Extraction Algorithm). We compare our PBEA with 19 other methods on the ISPRS Vaihingen and Toronto datasets. On the Vaihingen dataset, our PBEA achieves higher accuracy than the top five methods in terms of the area-based quality evaluation metric ranking list. On the Toronto datasets, our PBEA also achieves the highest accuracy, compared with the other top five methods in terms of the area-based quality evaluation metric ranking list. The two area-based quality evaluation metric ranking lists on the Vaihingen and Toronto datasets were sourced from the website of the ISPRS Test Project on Urban Classification and 3D Building Reconstruction [56]. In addition, our PBEA achieves higher overall accuracy than 10 state-of-the-art methods from the literature on the ISPRS Vaihingen dataset, which includes 4 deep-learning methods and 6 unsupervised methods. These comparison results demonstrate that our PBEA performs well across different datasets and is superior to the all methods compared on the area-based quality evaluation metric. Our PBEA can accurately and effectively extract large-scale buildings.

## Figures and Tables

**Table 1:** Performance comparisons of different snake models on the Vaihingen dataset.

Snake Models	Average Q
basic snake [45]	72.3%
GVF snake [46]	80.5%
T-snake [55]	83.3%
Nguyen snake [33]	86.5%
Our snake	89.1%

**Table 2:** Comparison results of different methods on the Vaihingen dataset in terms of the area-based evaluation metric.

Areas	Methods	C_p_-Are	C_r_-Are	Q-Are
Area 1	LJU2 [56]	**94.6%**	**9** **9.1%**	**93.8%**
	ZJU [56]	92.1%	95.2%	88.0%
	DLR [56]	91.9%	95.4%	88.0%
	CSU [56]	93.6%	94.5%	88.8%
	HKP [56]	92.0%	97.4%	89.8%
	Ours	93.5%	95.4%	89.5%
Area 2	LJU2 [56]	**95.1%**	94.3%	89.9%
	ZJU [56]	94.4%	97.7%	**92.3%**
	DLR [56]	94.3%	97.0%	91.6%
	CSU [56]	94.6%	95.4%	90.5%
	HKP [56]	93.3%	**98.4%**	91.9%
	Ours	94.3%	96.8%	91.4%
Area 3	LJU2 [56]	**94.6%**	95.6%	**90.7%**
	ZJU [56]	92.0%	96.3%	88.9%
	DLR [56]	93.7%	95.5%	89.7%
	CSU [56]	93.9%	94.7%	89.2%
	HKP [56]	89.2%	**97.7%**	87.4%
	Ours	92.6%	95.9%	89.0%
Average of three areas	LJU2 [56]	**94.8%**	94.3%	89.8%
	ZJU [56]	92.8%	96.4%	89.7%
	DLR [56]	93.3%	96.0%	89.8%
	CSU [56]	94.0%	94.9%	89.5%
	HKP [56]	91.4%	**97.8%**	89.6%
	Ours	93.4%	96.1%	**90.0%**

**Table 3:** Comparison results of different methods on the ISPRS Vaihingen dataset in terms of the object-based evaluation metric.

Areas	Methods	C_p_-Obj	C_r_-Obj	Q-Obj	C_p50m_^2^-Obj	C_r50m_^2^-Obj	Q_50m_^2^-Obj
Area 1	LJU2 [56]	**91.9%**	**100%**	**91.9%**	**100%**	**100%**	**100%**
	ZJU [56]	81.1%	**100%**	81.1%	**100%**	**100%**	**100%**
	DLR [56]	83.8%	96.9%	81.6%	**100%**	**100%**	**100%**
	CSU [56]	83.8%	**100%**	83.8%	**100%**	**100%**	**100%**
	HKP [56]	83.8%	**100%**	83.8%	**100%**	**100%**	**100%**
	Ours	84.6%	**100%**	84.6%	**100%**	**100%**	**100%**
Area 2	LJU2 [56]	**85.7%**	**100%**	**85.7%**	**100%**	**100%**	**100%**
	ZJU [56]	71.4%	90.9%	66.6%	**100%**	**100%**	**100%**
	DLR [56]	78.6%	**100%**	78.6%	**100%**	**100%**	**100%**
	CSU [56]	**85.7%**	**100%**	**85.7%**	**100%**	**100%**	**100%**
	HKP [56]	78.6%	91.7%	73.4%	**100%**	**100%**	**100%**
	Ours	80.1%	**100%**	80.1%	**100%**	**100%**	**100%**
Area 3	LJU2 [56]	83.9%	**100%**	83.9%	**100%**	**100%**	**100%**
	ZJU [56]	76.8%	**100%**	76.8%	97.4%	**100%**	97.4%
	DLR [56]	78.6%	**100%**	78.6%	**100%**	**100%**	**100%**
	CSU [56]	80.4%	**100%**	80.4%	**100%**	**100%**	**100%**
	HKP [56]	76.8%	97.8%	75.5%	97.4%	**100%**	97.4%
	Ours	**85.1%**	98.7%	**84.3%**	98.1%	**100%**	98.1%
Average of three areas	LJU2 [56]	**87.2%**	**100%**	**87.2%**	**100%**	**100%**	**100%**
	ZJU [56]	76.4%	97.0%	74.9%	99.1%	**100%**	99.1%
	DLR [56]	80.3%	99.0%	79.6%	**100%**	**100%**	**100%**
	CSU [56]	83.3%	**100%**	83.3%	**100%**	**100%**	**100%**
	HKP [56]	79.7%	96.5%	77.6%	99.1%	**100%**	99.1%
	Ours	83.3%	99.6%	83.0%	99.4%	**100%**	99.4%

**Table 4:** Rankings of the average evaluation metrics of the PEBA algorithm on the Vaihingen dataset.

Avg	Q-Are	RMSE	Q-Obj	Q_50m_^2^-Obj
Ranking	1	3	3	2

**Table 5:** Comparison with the state-of-the-art methods from the published literature.

Methods	Method Types	Data Types	Q-Are
Nguyen’s [33]	Unsupervised	LiDAR + Image	86.6%
Liu’s [28]	Unsupervised	LiDAR	87.7%
Dey’s [29]	Unsupervised	LiDAR	84.0%
Hui’s [30]	Unsupervised	LiDAR	88.5%
Mousa’s [37]	Unsupervised	DSM + Image	88.4%
Zhang’s [14]	Unsupervised	Image	89.3%
Protopapadakis’s SAFER [13]	Deep learning based	Image	82.9%
Protopapadakis’s WeiAve [13]	Deep learning based	Image	83.9%
Maltezos’s [38]	Deep learning based	LiDAR + Image	80.8%
Zhang’s [39]	Deep learning based	LiDAR + Image	87.3%
Ours	Unsupervised	LiDAR + Image	**90.0%**

**Table 6:** Comparison results of different methods on the ISPRS Toronto dataset in terms of the area-based evaluation metric.

Areas	Methods	C_p_-Are	C_r_-Are	Q-Are
Area 4	MAR1 [56]	95.2%	94.4%	90.1%
	MON2 [56]	95.1%	91.1%	87.0%
	MAR2 [56]	93.7%	94.9%	89.2%
	WHU_YD [56]	94.7%	**95.5%**	90.7%
	HKP [56]	96.6%	94.4%	91.4%
	Ours	**96.9%**	95.4%	**92.5%**
Area 5	MAR1 [56]	96.9%	89.7%	87.2%
	MON2 [56]	96.7%	93.2%	90.3%
	MAR2 [56]	94.3%	93.7%	88.7%
	WHU_YD [56]	96.9%	93.7%	91.0%
	HKP [56]	**98.6%**	91.0%	89.8%
	Ours	97.8%	**94.3%**	**92.3%**
Average of two areas	MAR1 [56]	96.1%	92.1%	88.7%
	MON2 [56]	95.9%	92.2%	88.7%
	MAR2 [56]	94.0%	94.3%	88.9%
	WHU_YD [56]	95.8%	94.6%	90.8%
	HKP [56]	**97.6%**	92.7%	90.6%
	Ours	97.4%	**94.8%**	**92.4%**

**Table 7:** Comparison results of different methods on the ISPRS Toronto dataset in terms of the object-based evaluation metric.

Areas	Methods	C_p_-Obj	C_r_-Obj	Q-Obj	C_p50m_^2^-Obj	C_r50m_^2^-Obj	Q_50m_^2^-Obj
Area 4	MAR1 [56]	**100%**	95.0%	95.0%	**100%**	96.6%	96.6%
	MON2 [56]	**100%**	83.6%	83.6%	**100%**	94.9%	94.9%
	MAR2 [56]	98.3%	94.9%	93.4%	**100%**	96.6%	96.6%
	WHU_YD [56]	98.3%	96.6%	95.0%	**100%**	96.6%	96.3%
	HKP [56]	98.3%	96.6%	95.0%	**100%**	96.6%	96.3%
	Ours	97.9%	**97.4%**	**95.4%**	**100%**	**96.9%**	**96.9%**
Area 5	MAR1 [56]	**97.4%**	78.6%	77.0%	**97.1%**	78.6%	76.8%
	MON2 [56]	86.8%	78.6%	70.2%	91.4%	94.1%	86.4%
	MAR2 [56]	84.2%	88.9%	76.2%	91.4%	**97.0%**	**88.9%**
	WHU_YD [56]	84.2%	**94.1%**	80.0%	91.4%	94.1%	86.4%
	HKP [56]	89.5%	84.2%	73.5%	91.4%	84.2%	78.0%
	Ours	90.3%	89.6%	**81.7%**	92.6%	93.7%	87.2%
Average of two areas	MAR1 [56]	90.8%	86.8%	79.8%	89.3%	87.6%	78.5%
	MON2 [56]	93.4%	81.1%	76.7%	95.7%	94.5%	90.7%
	MAR2 [56]	91.3%	91.9%	84.5%	95.7%	**96.8%**	**92.8%**
	WHU_YD [56]	91.3%	**95.4%**	87.4%	95.7%	95.4%	91.4%
	HKP [56]	93.9%	90.4%	85.4%	95.7%	90.4%	86.9%
	Ours	**94.1%**	93.5%	**88.5%**	**96.3%**	95.3%	92.1%

**Table 8:** Rankings of average evaluation metrics of our PEBA on the Toronto dataset.

Avg	Q-Are	RMSE	Q-Obj	Q_50m_^2^-Obj
Ranking	1	1	1	2

## Data Availability

Data are contained within the article.
